# EVALUATION OF SMART BELT HIP PROTECTION IN A SKILLED NURSING FACILITY

**DOI:** 10.1093/geroni/igz038.3373

**Published:** 2019-11-08

**Authors:** Rebecca J Tarbert, Wamis Singhatat

**Affiliations:** Active Protective Technologies, Inc, Fort Washington, Pennsylvania, United States

## Abstract

Hip fractures impact > 300,000 US older adults yearly resulting in 70,000 deaths and are expected to gain in numbers with the rising population. Residents of skilled nursing facilities are among the highest at risk of sustaining hip fractures due to increased risk of falls and frailty. These most vulnerable stand to experience the most serious results of hip fracture with > 50% resulting in total dependency and/or death. Care providers are focused on providing an environment of safety with implementation of traditionally utilized fall prevention measures. Unfortunately, the maintenance of safety in this high-risk population often comes at the price of limiting independent mobility. The utilization of passive hip protector padding for those recognized as being at high risk of hip fracture can decrease the risk of hip fracture by 82%; however, challenges to adherence of hip protectors limit the effectiveness of this widely utilized measure. Emerging technology in the form of a smart belt was evaluated in a skilled nursing setting to offer insight into efficacy and user adherence. The smart belt is capable of sensing when the wearer is experiencing a motion that would likely result in a fall onto the hip, deploy an anatomically conforming airbag and alert caregivers that a fall has occurred. The embedding of the hip protection technology into care planning led to daily patient utilization totaling over 3000 hours. Specific findings of the user derived motion and experience will be articulated through case studies and illustration of the technology captured motion data.

